# Supplementary figures and images for: A Caenorhabditis elegans nck-1 and filamentous actin-regulating protein pathway mediates a key cellular defense against bacterial pore-forming proteins
Source: PLoS Pathog. 2022 Nov 14;18(11):e1010656. doi: 10.1371/journal.ppat.1010656 (PMC9704757; doi:10.1371/journal.ppat.1010656)

**S1 Table**


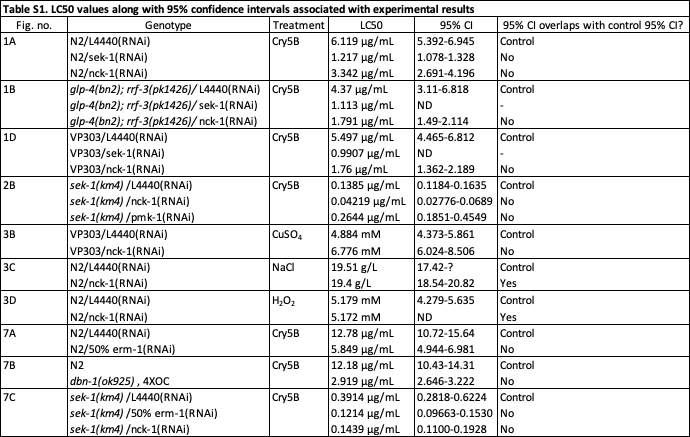

Supplement: S1 Table — (DOCX) [file ppat.1010656.s001.docx]

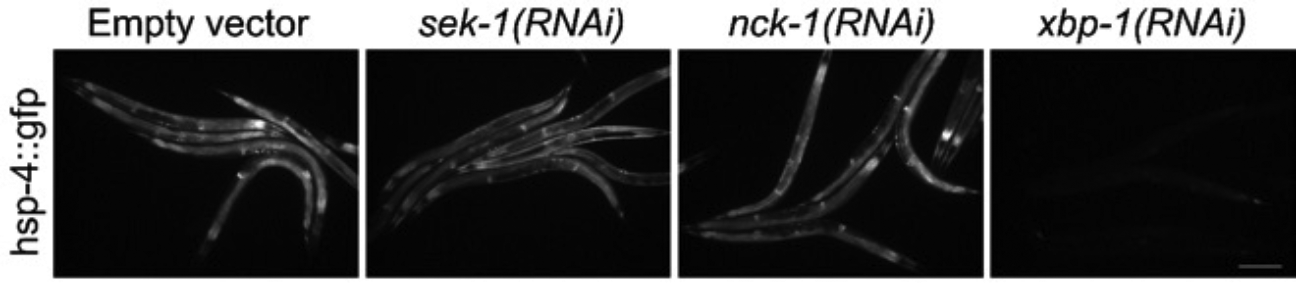

Supplement: S1 Fig — hsp-4::gfp worms grown on the indicated RNAi bacteria to the L4 stage, moved to 30°C for heat shock, and then photographed after 8 hours of incubation at that temperature. Scale bar = 0.1mm. (TIF) [file ppat.1010656.s002.tif]

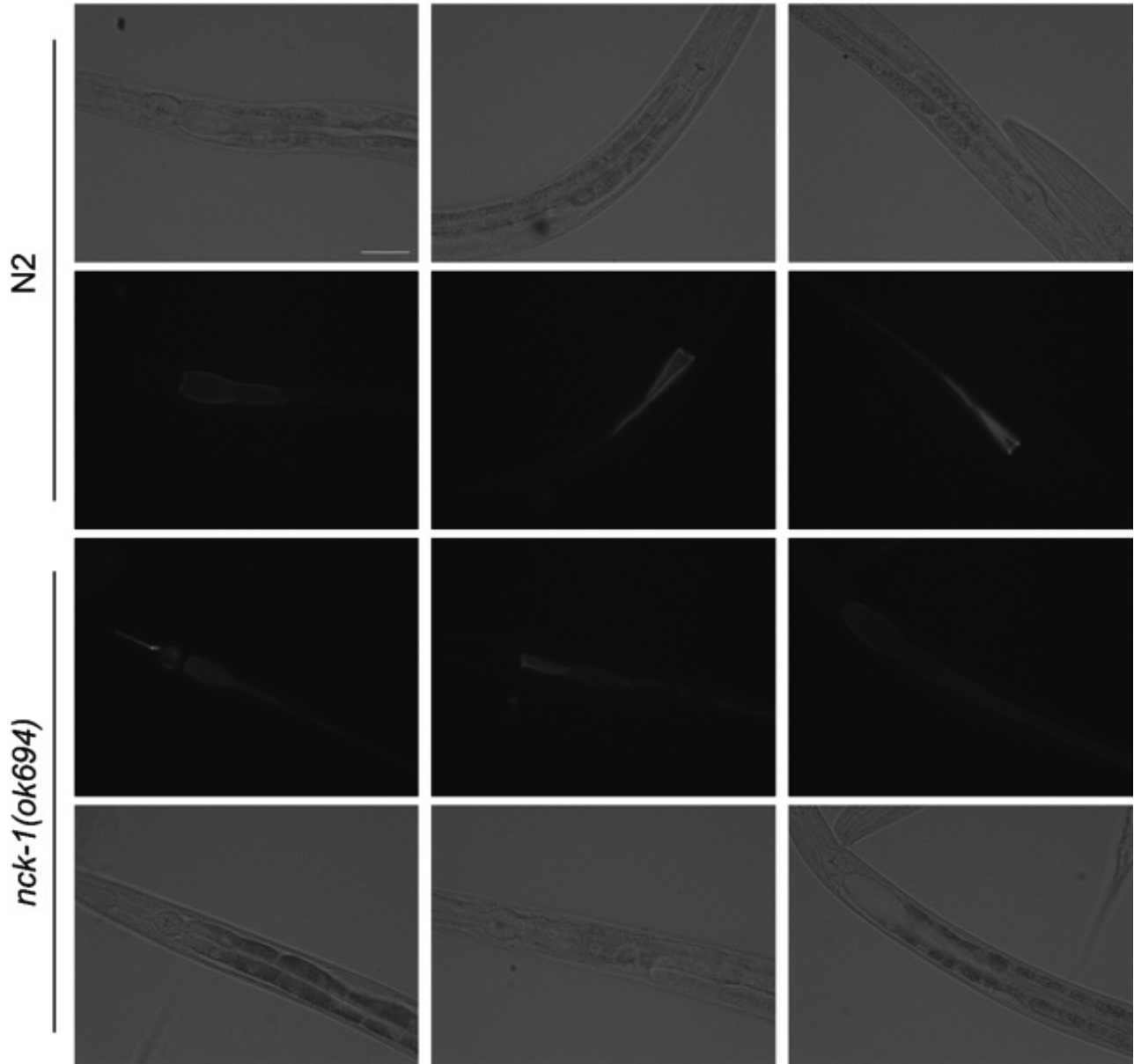

Supplement: S2 Fig — The indicated C. elegans strains were grown to the L4 stage and subjected to the normal propidium iodide feeding protocol, with no exposure to Cry5B. Scale bar = 25um. (TIF) [file ppat.1010656.s003.tif]
